# Fully Bio-Based Polymer Composites: Preparation, Characterization, and LCD 3D Printing

**DOI:** 10.3390/polym16091272

**Published:** 2024-05-02

**Authors:** Giovanna Colucci, Francesca Sacchi, Federica Bondioli, Massimo Messori

**Affiliations:** 1 Politecnico di Torino, Department of Applied Science and Technology (DISAT), Corso Duca degli Abruzzi 24, 10129 Torino, Italy; francesca.sacchi@polito.it (F.S.); federica.bondioli@polito.it (F.B.); massimo.messori@polito.it (M.M.); 2 National Interuniversity Consortium of Materials Science and Technology (INSTM), Via G. Giusti 9, 50121 Firenze, Italy

**Keywords:** bio-based polymer composites, acrylate epoxidized soybean oil, 3D printing, liquid crystal display, biofillers

## Abstract

The present work aimed to prepare novel bio-based composites by adding fillers coming from agro-wastes to an acrylate epoxidized soybean oil (AESO) resin, using liquid crystal display (LCD) 3D printing. Different photocurable formulations were prepared by varying the reactive diluents, iso-bornyl methacrylate (IBOMA) and tetrahydrofurfuryl acrylate (THFA). Then, two fillers derived from different industrial wastes, corn (GTF) and wine (WPL-CF) by-products, were added to the AESO-based formulations to develop polymer composites with improved properties. The printability by LCD of the photocurable formulations was widely studied. Bio-based objects with different geometries were realized, showing printing accuracy, layer adhesion, and accurate details. The thermo-mechanical and mechanical properties of the 3D-printed composites were tested by TGA, DMA, and tensile tests. The results revealed that the agro-wastes’ addition led to a remarkable increase in the elastic modulus, tensile strength, and glass transition temperature in the glassy state for the systems containing IBOMA and for flexible structures in the rubbery region for systems containing THFA. AESO-based polymers demonstrated tunable properties, varying from rigid to flexible, in the presence of different diluents and biofillers. This finding paves the way for the use of this kind of composite in applications, such as biomedical for the realization of prostheses.

## 1. Introduction

Recently, several 3D-printing processes for polymers have been commercially introduced to the market, and a growing number of 3D printers for academic, industrial, and home use are extending the use of additive manufacturing (AM) processes, as consumers can design objects based on their desires [1,2]. AM technologies show many advantages with respect to the traditional manufacturing methodologies [1,2,3,4,5]. In fact, they lead to the realization of components and prototypes with complex geometry and high design flexibility and to the creation of functional parts without the need for assembly, reducing time, costs, and waste. Moreover, they are solvent-free methods, no additional tools or molds are required, and the material not used for printing can be recycled and reused for the realization of other jobs [1,2,3,4,5]. For this reason, AM can be considered an environmentally friendly approach to the realization of parts, which can find applications in many different industrial fields. The automotive, aerospace, and medical fields are the three main sectors that have exploited the application of AM technologies for designing and developing structural components such as engine exhausts; for the production of highly complex internal functional parts, such as turbines; or, again, for the manufacture of complex geometrical structured parts, including orthopedic and dental implants, tissue scaffolds, and prostheses [6,7]. Among all the AM techniques, VAT photopolymerization (VP) represents a suitable method for printing, in an easy way, small and dimensionally accurate objects with specific geometries using liquid photopolymers. It is a light-based AM technology, which allows for the obtainment of the highest printing resolution and shape complexity [8,9]. During the printing process, the build platform is dipped into the liquid resin and, thanks to the UV light, the photocurable resin in the vat can be transformed into a uniform layer of solid material according to the previously designed CAD model. The process is repeated continuously until the object is complete [8,9]. Vat polymerization technologies are generally divided into different categories based on the light source used on the 3D printer. These are mainly laser-based stereolithography (SLA), direct light processing (DLP), and liquid crystal display (LCD), also called daylight polymer printing (DPP). The key idea of the last type of process, LCD, is to manufacture the desired component by gradually building up the part using a layer-by-layer approach starting from a liquid formulation and resulting in the selective curing of the thermoset polymer [8,9,10]. LCD involves a bottom-up configuration, where the print platform is located inside the vat, completely immersed into the liquid resin, and the light is emitted from the bottom, polymerizing the resin on the platform surface by means of an LED lamp given by an LCD screen. When one layer is finished, the platform is slightly raised, and the process is repeated to create the next layer. The process continues until the object is completed [10,11]. Other advantages of the LCD process are the wide family of usable thermosetting polymers and the possibility of testing a combination of chemical compounds and reagents, and thus customizing photopolymers by trying to design and produce parts by modifying material properties or creating new materials. Moreover, it involves the low consumption of raw materials while achieving high-resolution products by using a green approach. However, despite these advantages, the applicability of LCD is strictly limited due to the low number of sustainable photopolymerizable resins available on the market [10,11]. To accelerate the transition towards a circular economy, the development of innovative bio-based resins for 3D printing can play a key role. In this research framework, vegetable oils are the most widely used materials, because they are abundant in nature, renewable, inexpensive, and biodegradable, and because they exhibit structural features that make them very attractive for a wide range of applications [12]. Many studies regarding the use of soybean oil in vat photopolymerization have been published. They found that vegetable oils have excellent properties if applied to 3D-printing processes based on the photopolymerization reaction [12,13,14,15]. However, pure soybean oil resin does not exhibit sufficient mechanical properties for engineering applications. Thus, resin is generally combined with fillers or fibers to obtain polymer composites useful in many application fields like the agricultural, automotive, infrastructure, or biomedical sectors due to the improved mechanical properties [12,13,14,15]. Many authors prepared soybean oil-based composites reinforced with functionalized calcium silicate [16], cellulose [8], vanillin [17], or particles of macadamia nutshells [18] by DLP for mechanical properties’ improvements. Other authors used AESO for the realization of composites with enhanced electrical properties by 3D printing [19] or, again, biocompatible scaffolds for medical applications, such as human bones, tissue engineering, or prostheses [20,21,22].

Although the literature presents a wide collection of studies on 3D-printed polymer composites, the addition of bio-based fillers derived from agro-wastes to soybean oil resin for the realization of composites by LCD [23,24] has been poorly investigated if compared to other techniques, like DLP.

For this reason, the aim of the present research highlights the realization of biodegradable soybean oil-based parts by using LCD as a 3D-printing technique.

This paper aims to combine the advantages of bio-based composites prepared by valorizing biofillers coming from agricultural industry wastes and the advantages of 3D-printing vat photopolymerization. It focuses attention on the preparation and characterization of polymer composites by using a relatively new VP technique like LCD, which gives rise to the realization of 3D-printed components with a higher print quality and accuracy with respect to DLP 3D printing. This can prevalently be due to the density of pixels of the LCD screen resolution, which offers the opportunity to print larger components with no difference in terms of quality from smaller models [24]. Moreover, LCD 3D printers are more versatile and less expensive, even if the required print speed is usually slower than DLP. Furthermore, in this 3D-printing technique, a wide range of photocurable resins can be used, providing users more flexibility in choosing materials for different applications, including prototypes and functional parts [23,24]. The viscosity of the photopolymers plays a key role in the LCD-printing process. This means that the resin viscosity needs to be carefully controlled to ensure proper resin flowability, good layer formation, and curing during the printing process [24].

For this reason, two bio-based monofunctional diluents, iso-bornyl methacrylate (IBOMA) and tetrahydrofurfuryl acrylate (THFA), were firstly added to AESO resin in order to tune its viscosity and increase its flowability. The formulations were polymerized by an LCD 3D printer to obtain samples as references. Then, AESO-based composites were prepared by dispersing two different kinds of fillers coming from industrial wastes within the photocurable matrix to enhance its final properties.

## 2. Materials and Methods

### 2.1. Materials

Acrylate epoxidized soybean oil (AESO) was used as a bio-based starting photocurable resin. It is as a viscous yellow liquid, with a density of 1.04 g/mL at 25 °C. Two different bio-based diluents were employed to prepare the polymeric photocurable formulations: iso-bornyl methacrylate (IBOMA), coming from pine trees with a density of 0.983 g/mL at 25 °C, and tetrahydrofurfuryl acrylate (THFA) with a density of 1.064 g/mL at 25 °C. Phenyl bis (2,4,6-trimethylbenzoyl) phosphine oxide (BAPO) was chosen as a radical photoinitiator. All the chemicals for the formulations were purchased from Merck (Merck KGaA, Darmstadt, Germany.

Two different biofillers coming from agro-wastes were added to the photocurable formulations: a yellow natural filler derived from maize by-products, named GTF, and a purple powder obtained by pressing grape solid residues obtained from wine production, named WPL-CF, kindly supplied by Agromateriae srl (Faenza, RA, Italy).

### 2.2. Bio-Based Composites Preparation

Several photocurable formulations were firstly prepared by mixing the acrylate epoxidized soybean oil resin (AESO) with different weight percentages of the two selected bio-based diluents, IBOMA and THFA, in a range between 20 to 50 wt.%, in the presence of BAPO as a photoinitiator for each formulation.

The prepared photocurable formulations were then tested in terms of viscosity and flowability to determine the best compromise between AESO resin viscosity and the diluent capacity of IBOMA or THFA, which can guarantee good final printability for LCD 3D-printing processing.

After the preliminary tests, once the best AESO-based formulation in terms of viscosity and processability had been identified, the formulations containing 60 wt.% of AESO and 40 wt.% of IBOMA or THFA were selected. Subsequently, bio-based composites were prepared by adding 2 parts per hundred resin (phr) of BAPO as a photoinitiator and 5 phr of the two biofillers (GTF and WPL-CF) derived from agricultural wastes to the liquid photocurable formulations.

The dispersion of the fillers was carried out by combining 30 min of magnetic stirring and 15 min of an ultrasonic bath for a total of 4 h to promote a faster dispersion of the powders within the photocurable mixture containing AESO resin and diluents. This procedure made the process less time-consuming and avoided the formation of air bubbles, which can have negative effects on the following printing process.

### 2.3. 3D Printing of AESO-Based Samples by LCD 3D Printer

AESO-based formulations were 3D printed by using a Phrozen Sonic Mini 8K (Phrozen, Hsinchu City, Taiwan) vat photopolymerization 3D printer, LCD type. This printer is characterized by a Linear Projection LED Module, which has an xy resolution of 22 μm, while it has a z resolution or layer thickness e between 0.01 and 0.30 mm and a printing volume of 16.5 (l) × 7.2 (w) × 18 (h) cm^3^. Moreover, the platform size of this 3D printer is very small, with a volume of 29 (l) × 29 (w) × 43 (h) cm^3^, and it allows the use of a very low quantity of photocurable resins.

The LCD 3D printing machine is also equipped with a slicer software, called CHITUBOX V1.9.0, which permits the STL file of the desired part to be loaded, the orientation of the piece to be chosen, and, eventually, for supports that can connect the piece to the printing platform to be applied. The program also allows us to modify the printing parameters, like layer thickness, exposure time, and speed, during the printing process.

Firstly, the printability of each AESO-based formulation was studied, and the corresponding printing parameters were optimized for the preparation of the composite formulations. The 3D-printed samples, removed from the platform, were washed in iso-propyl alcohol (Merck) and post-cured for 30 min with an Anycubic Wash & Cure Plus machine (Anycubic Technology Co, Hongkong).

### 2.4. Characterization Techniques

The viscosity of AESO-based liquid formulations containing the two different diluents was measured at 25 °C by using an Anton Paar MCR 702e MultiDrive Rheometer (Graz, Austria) in a parallel plate configuration and in the shear range of 10–104 L/s.

The insoluble fraction, also known as gel content, of rectangular photocured samples was determined by measuring the weight loss of each sample after 24 h of extraction with chloroform at room temperature, according to the ASTM D2765-84 technical standard.

The morphology of the specimens was investigated by a Phenom™ XL Scanning Electron Microscope (Thermo Fisher Scientific, Waltham, MA, USA) at a voltage of 10 kV. The samples were previously metallized with platinum for 15 s using a Quorum Sputter Coater, Q150T S (Laughton, East Sussex, UK). Each specimen was fractured in liquid nitrogen and the fracture surface was analyzed.

Thermogravimetric (TG) analysis was performed by using a Mettler-Toledo TGA 851e instrument (Columbus, OH, USA). All the 3D-printed samples were heated from 25 up to 900 °C at a heating rate of 10 °C/min in air. The TG curves were normalized to the mass of each sample, and the DTG curves were calculated on their relative thermograms.

The dynamic mechanical analysis (DMTA) was carried out from 0 to 200 °C by using an Anton Paar MCR 702e Multi Drive Rheometer (Graz, Austria) at a frequency of 1 Hz in tensile configuration. The samples tested were rectangular with a length (l) of 50 mm, a width (w) of 10 mm, and a thickness (t) of 2 mm.

The mechanical properties of the 3D-printed sample, such as Young’s modulus, tensile strength, and elongation at break, were investigated by tensile tests according to ASTM D638-14, using an Instron 5966 equipped with a 2 kN load cell and pneumatic grips, with a deformation rate of 5 mm/min and a grip separation of 50 mm. Deformation was calculated using a displacement transducer. The specimens were type 5 and dog bone-shaped, with gauge lengths of 26 mm, widths of 4 mm, and thicknesses of 2 mm.

## 3. Results and Discussion

### 3.1. Biofillers’ Characterization

**Figure 1:**
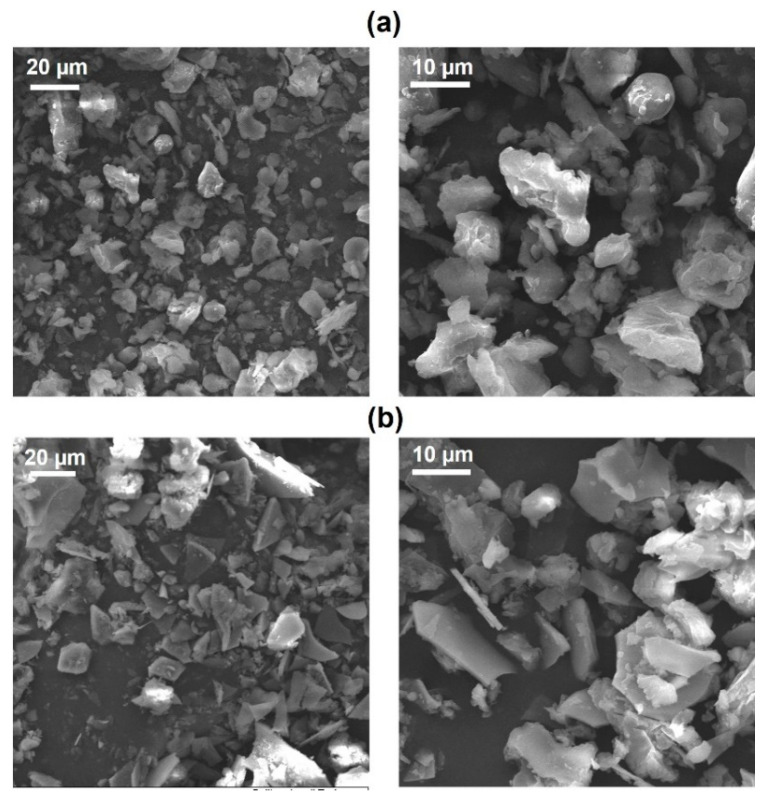
SEM micrographs at different magnifications of GTF (**a**) and WPL-CF (**b**).

The two biofillers coming from agro-wastes were characterized from morphological and thermal points of view. SEM images, reported in Figure 1, were firstly acquired to examine the morphology of the GTF (a) and WPL-CF (b) fillers, which are characterized by the typical structure of milled materials with average dimensions between 20 and 40 µm. GTF powder has tiny flake particles, while WPL-CF presents a different morphology with more irregular-shaped particles, probably due to its origin from wine seeds.

**Figure 2:**
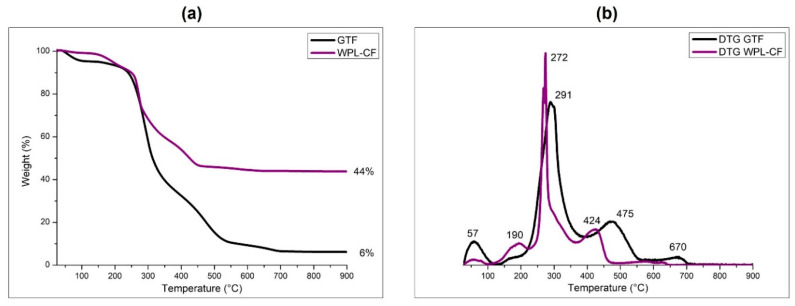
TG (**a**) and DTG (**b**) curves of GTF and WPL-CF powders, performed in air.

Figure 2 presents the TG (a) and the first derivative curves (b) for the two agro-wastes, GTF (black curve) and WPL-CF (purple curve). As is visible, the fillers show quite similar thermal behavior. The early weight loss was found at low temperature, 57 °C, and can be attributed to the desorption of moisture eventually absorbed on the biofillers’ surfaces, which is more evident for GTF.

This can probably be explained by the fact that this filler is obtained by reusing corn wastes, which contain higher fractions of cellulose [25]. In both the cases, a very broad pattern in the range of temperatures where the organic fractions of GTF and WPL-CF start to thermally degrade is evident. A large part of the fillers starts to degrade between 200 to 400 °C, and this can be ascribed to the degradation of the hemicellulose and cellulose fractions, with a main degradation peak at 291 °C followed by a second degradation peak at 475 °C for GTF.

WPL-CF powder starts to degrade at a lower temperature, 190 °C, and shows a first degradation peak at 272 °C and a second peak at 424 °C. However, the major difference in the thermal behavior of the two fillers is related to the residue content at 900 °C. GTF powder shows a residue of 6 wt.%, whereas for WPL-CF, a higher residue of 44 wt.% is found at the same temperature.

This can be attributed to the different origins of the two agro-waste fillers. GTF is derived from corn by-products, while WPL-CF is obtained from seed waste in wine production, which contains higher amounts of inorganic compounds.

### 3.2. AESO-Based Photocurable Formulations

AESO-based mixtures with biodegradable diluents, such as IBOMA and THFA, were first prepared by LCD 3D-printing technology. Then, the two different agro-wastes were added to the photocurable AESO-IBOMA and AESO-THFA mixtures at concentrations of 5 phr. All the tested AESO-based formulations are listed in Table 1. Before proceeding with the 3D-printing process of the AESO-based formulations, their viscosity was tested to evaluate the effect of the addition of reactive diluents to the resin flowability, which can significantly influence their final printability.

**Figure 3:**
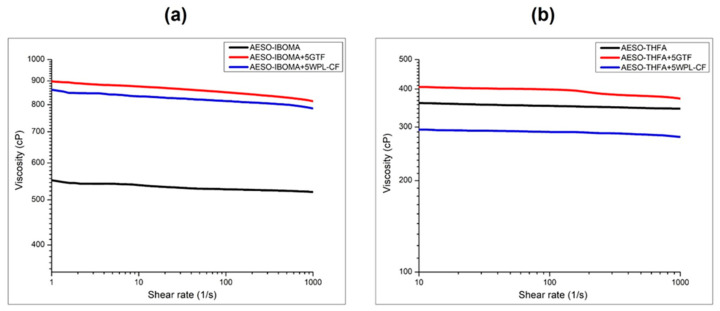
Rheograms (viscosity vs. shear rate) of AESO-based liquid formulations based on IBOMA (**a**) and THFA (**b**).

The results of viscosity tests of the AESO-based formulations containing IBOMA and THFA and of the composites obtained by adding 5 phr of the two agro-wastes are reported in Figure 3.

Figure 3a presents the rheograms of the unfilled and filled photocurable AESO-based formulations containing IBOMA. As expected, the zero-shear viscosity ($\eta_{0}$) of AESO-IBOMA mixtures in the presence of agro-wastes strongly increases from 535 cP to 860 and 830 cP for systems containing GTF and WPL-CF.

The values of viscosity obtained for samples containing IBOMA are considered useful for printable resin in the vat photopolymerization 3D-printing process. In fact, as reported in the literature, the optimal viscosities of a photocurable resin are usually set below 1000 cP [26].

On the contrary, different behavior can be observed for samples obtained by dispersing the fillers within the AESO matrix in the presence of THFA as a diluent.

Figure 3b shows the values of zero-shear viscosity of the unfilled and filled AESO-THFA formulations, revealing that, for this kind of system, the viscosity values remain almost in the same range, being in any case acceptable for vat photopolymerization 3D printing.

The values of zero-shear viscosity increase from 355 cP to 400 cP for composites obtained by adding 5 phr of GTF. Indeed, a slight reduction in terms of viscosity can be observed for systems loaded with WPL-CF, from 355 to 290 cP, even if the photocurable formulations show a good dispersion and distribution of the fillers without any visible sedimentation. As is known from the literature, the viscosity range of photopolymers for LCD 3D printing typically falls in a medium-viscosity range (i.e., 100–500 cP). These viscosity values allow the resin to flow easily within the vat and spread over the platform or previous layers, requiring higher curing times, and reaching a balance between its flowability and printability. [26,27]. All the AESO-based formulations were stable and well dispersed with no phase separation, particle deposition, or agglomerations observed.

### 3.3. 3D Printing of AESO-Based Liquid Formulations by LCD

Several preliminary printing tests were carried out to define the processing parameters and to assess the printability of the AESO-based resins with an LCD printer, guaranteeing the optimization of the photocuring process for the resins. The parameters optimized for the realization of the AESO-based formulations are summarized and listed in Table 2.

Furthermore, the presence of the biofillers led to an increase in the printing times and a decrease in the speed. This can be surely ascribed to the dye effect induced to the yellow (GTF) and purple (WPL-CF) powders introduced into the photocurable formulations [28]. Moreover, there is no difference to underline between the parameters used for composites prepared with the same diluent but with different types of filler.

**Figure 4:**
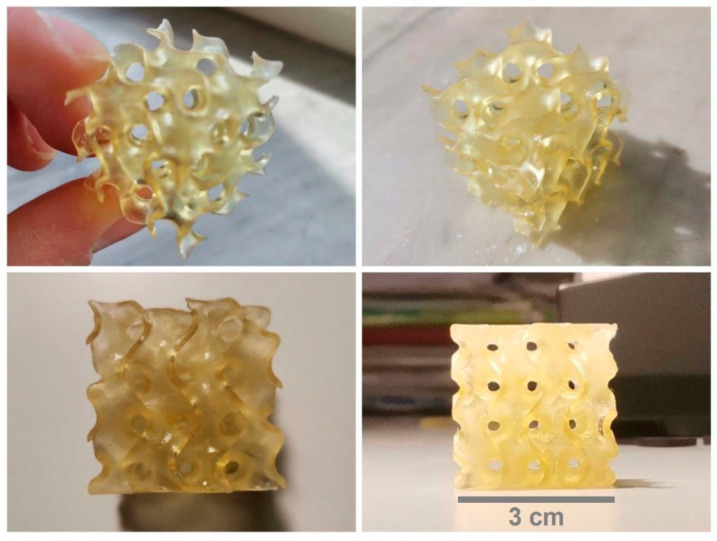
Gyroid cube-shaped 3D-printed sample based on AESO-IBOMA.

**Figure 5:**
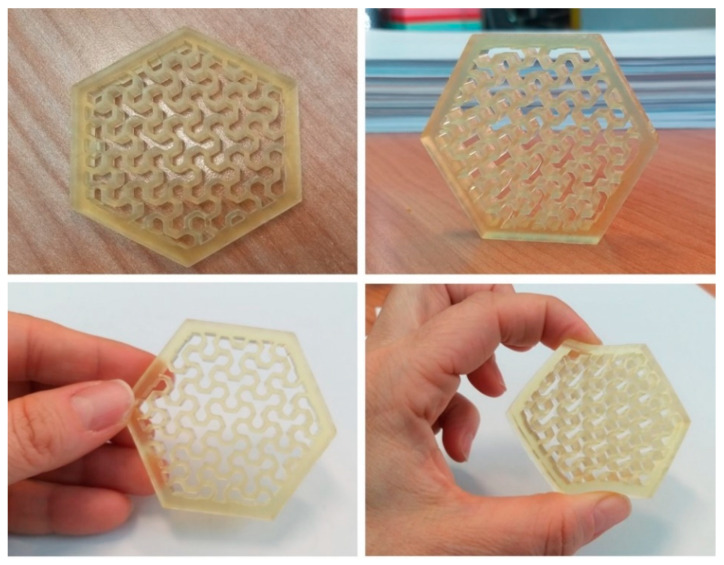
Hexagonal-shaped, 3D-printed sample based on AESO-THFA.

Having found the appropriate printing parameters for each formulation, different AESO-based polymer composite specimens were 3D printed, starting with simple geometry, like specimens rectangular in shape or dog bone shaped, up to more complex structures, like a gyroid cube reported in Figure 4 and a hexagon with singular internal pattern shown in Figure 5.

The sample shown in Figure 4 is rigid and shows a very good level of definition and a detail-rich shape, even on a very small scale. The specimen was successfully realized by the deposition of 300 layers of the AESO-IBOMA formulation. An example of a 3D-printed part obtained by adding THFA as diluent to the AESO matrix is shown in Figure 5, representing a complex hexagonal structure with a particular internal design where curvatures alternate full spaces. The hexagon is characterized by a very flexible structure, due to the intrinsic properties of THFA, and good, accurate print quality. It was obtained using LCD by depositing 80 layers of AESO-THFA.

**Figure 6:**
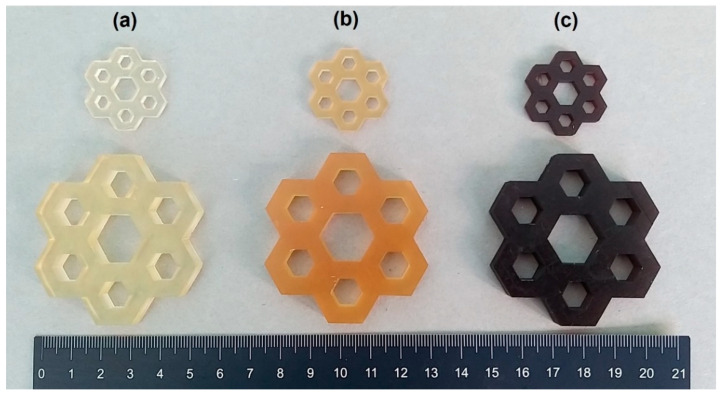
Three-dimensionally printed AESO-based samples with different dimensions: (**a**) AESO-IBOMA, (**b**) AESO-IBOMA+5GTF, and (**c**) AESO-IBOMA+5WPL-CF.

**Figure 7:**
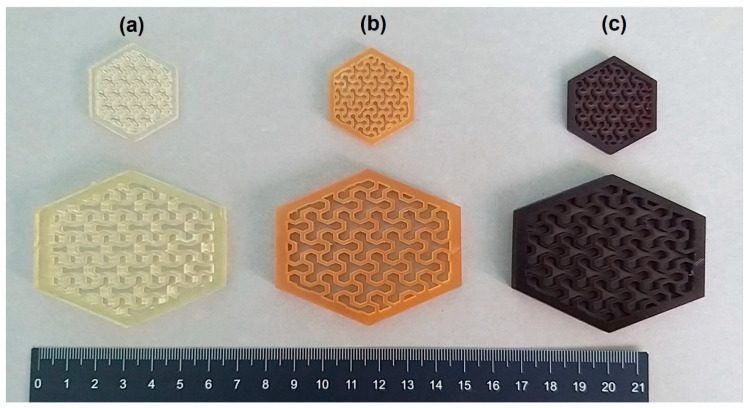
Three-dimensionally printed AESO-based samples with different dimensions: (**a**) AESO-THFA, (**b**) AESO-THFA+5GTF, and (**c**) AESO-THFA+5WPL-CF.

Figure 6 and Figure 7 show pictures of specimens realized via LCD after the addition of the two different agro-wastes (GTF and WPL-CF) to the photocurable AESO-based formulations containing IBOMA and THFA, respectively. The pictures in Figure 6 represent typical hexagonal honeycomb-like cells made by the deposition of 4 and 80 layers of the AESO-based resin in the presence of IBOMA as a diluent (a) and of the relative composites filled with 5 phr of GTF (b) and WPL-CF (c). The samples reveal good dimensional and printing accuracy, also evidencing a significant change in color due to the presence of the fillers, which act as natural yellow (b) and purple (c)colorants, respectively.

Figure 7 shows examples of objects realized by LCD by adding the two agro-wastes at 5 phr within the AESO-based formulation containing THFA as the diluent. The specimens represent hexagons with intricate internal patterns with hollowed and thin interconnected walls, made by 4 and 80 layers of photocured resin (a). At the same time, Figure 7 illustrates hexagonal-shaped structures realized by photocuring AESO-based formulations containing THFA filled with the two kinds of agro-wastes, GTF (b) and WPL-CF (c). The 3D-printed objects realized even using an LCD 3D printer for the composites show high printing accuracy of small details and well-defined coloration induced to the presence of the biofillers, which can act as dye agents.

### 3.4. Insoluble Fraction

Photocured rectangular specimens showed high gel content after 24 h of extraction in chloroform at room temperature, with values of 99.5 and 99.9 wt.% for the AESO samples with IBOMA and THFA, respectively. The obtained results (Table 3) underline that the gel content values slightly decrease with the addition of biofiller particles. The results indicate that the amount of extractable monomers is very low, i.e., most of the reactive monomers are strongly linked to the photocured polymer network [14]. This provided evidence that the optimized printing parameters are valuable for obtaining very good photopolymerization conversion of the resin monomers into an LCD 3D printer.

### 3.5. Microstructure of AESO-Based Composites

**Figure 8:**
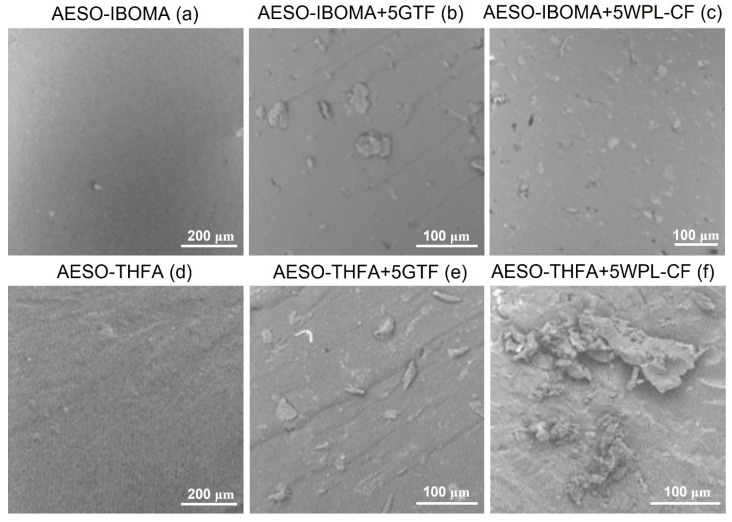
Three-dimensionally printed AESO-based specimens’ micrographs: (**a**) AESO-IBOMA, (**b**) AESO-IBOMA+5GTF, (**c**) AESO-IBOMA+5WPL-CF, (**d**) AESO-THFA, (**e**) AESO-THFA+5GTF, and (**f**) AESO-THFA+5WPL-CF.

The microstructure of the AESO-based composites realized by LCD was investigated by means of SEM (Figure 8). The analysis of the fracture surfaces of the AESO-based composites obtained by adding agro-wastes into the AESO resin revealed a better dispersion of the filler particles within the matrix in the case of the AESO-IBOMA system with respect to AESO-THFA.

In fact, the micrographs of Figure 8b,c show a uniform distribution and dispersion of the particles with the typical flake shape and good adhesion between the fillers and the matrix, as the fillers were still embedded in the crosslinked polymer matrix.

A certain number of agglomerates can be noticed, instead, for the composites obtained by dispersing the two biofillers into the AESO-THFA formulations. The presence of regions with micro-clusters of flakes is clear, particularly for composites loaded with WPL-CF, as reported in Figure 8e,f. This can be attributed to the higher time of exposure employed to print the series of composites containing THFA as diluent, due to its lower capacity to reduce the AESO viscosity (Table 1).

### 3.6. Thermal and Viscoelastic Properties

**Figure 9:**
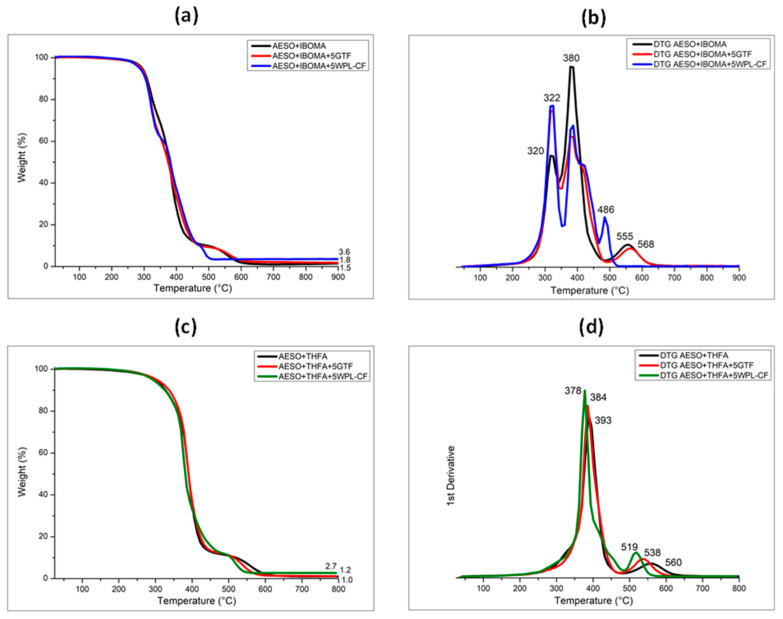
TG and DTG curves of 3D-printed samples containing IBOMA (**a**,**b**) and THFA (**c**,**d**) unfilled and filled with agro-wastes.

The thermal and viscoelastic properties of the AESO-based photocured samples and the relative composites were evaluated by means of TG and DMA analyses. Figure 9 presents the TG thermograms of the samples obtained by adding IBOMA (a) and THFA (b) as diluents and 5 phr of biofillers within the AESO matrix. The thermograms of the different systems present almost the same profile to underline that the presence of fillers did not impact the sample thermal stability, independently from the AESO/diluent mixture, as has already been reported [29,30].

The TG curves of the photocured AESO-IBOMA resins showed two-stage degradation peaks, at around 320 and 380 °C, due to the scission of the ester bond of the iso-bornyl group [14], as reported in Figure 9a,b. This also causes the presence of the lowest value of ash content (1.5%). Meanwhile, the photocured AESO-THFA resins reveal single-step degradation with a maximum peak fixed at around 393 °C, characteristic of the furan ring moieties of the THFA. Moreover, the degradation peaks related to the agro-wastes seen in Figure 2 are no longer evident because of the low amount of fillers. In fact, they are completely covered by the degradation peaks of the resin mixed with the reactive diluents, as shown in Figure 9c,d. The onset degradation temperatures (Tonset), obtained by analyzing TG thermograms, and the maximum degradation peaks (Tmax deg peak), obtained by DTG curves, are listed and summarized in Table 3.

A significant difference between the two series of samples (AESO-IBOMA and AESO-THFA) can be found in the ash yield values, detected at 900 °C of the TG curves. If the addition of GTF led to a slight increase in the ash content percentage for both the systems, on the contrary, the dispersion of WPL-CF within the AESO-based resin induced a remarkable increase in the ash yield, as expected when evaluating the results obtained from the pure agro-waste (Figure 2).

In particular, the ash content percentage shifted from 1.5 to 3.6% for composites containing AESO+IBOMA and from 1.0 to 2.7% for those containing AESO+THFA (Table 3).

**Figure 10:**
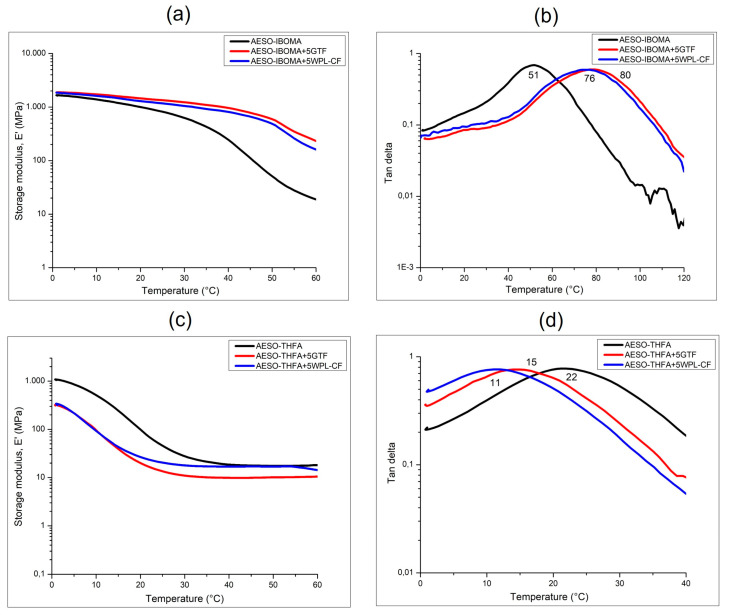
DMA curves of 3D-printed samples containing IBOMA (**a**,**b**) and THFA (**c**,**d**) with and without biofillers.

Furthermore, Figure 10 reports the comparison between the DMA curves of the crosslinked AESO-based samples containing IBOMA (a and b) and THFA (c and d) in the presence of the two biofillers. The unfilled samples presented completely different storage modulus values, 1633 MPa and 1040 MPa, respectively, for AESO-IBOMA and AESO-THFA, that once again underline the different effects induced to the reactive diluents to the AESO matrix. In fact, if IBOMA leads, at room temperature, to the formation of a rigid structure in the glassy state, THFA at room temperature gives rise to more flexible structures staying the rubbery region, as already seen for the 3D-printed samples reported in Figure 6 and Figure 7.

Meanwhile, the presence of the fillers within the AESO-IBOMA photocurable formulations allowed the elastic modulus to be enhanced in a significant way. In fact, the E’ values, determined in the glassy state (0 °C), shifted from 1633 MPa to around 1850 MPa, probably due to the stiffening effect on the polymeric chains induced to the filler particles [31]. Completely different behavior could be observed for the composites prepared by dispersing the biofillers within the AESO-THFA mixture. The storage modulus values decreased from 1040 MPa to about 310 MPa, and this could be attributed to the biofiller particles’ presence, resulting in lower mechanical properties at temperatures below the Tg. This difference was not evident at temperatures higher than the glass transition temperature in the rubbery plateau [32].

DMA analysis also allowed us to achieve the exact monitoring of Tg values as maximum peaks of tan delta curves. A considerable increase in the Tg (about 30 °C; see Table 3) was achieved for AESO-IBOMA composites due to the reinforcement effect induced by the presence of the biofillers, which led to restrictions of the polymeric chain motions. Moreover, the increase in the glass transition towards higher temperatures underlined the formation of a more rigid structure. On the contrary, a decrease in Tg values (Table 3) was observed for the composites obtained starting from AESO-THFA photocurable samples, as already seen for the storage modulus values.

This can be explained by considering the flexibility effect due to the biofiller particles on the polymer matrix mixed with THFA. Furthermore, it is possible to see that the tan delta curves exhibited a broader profile indicative of poor adhesion between the layers occurring during the printing process [32,33]. As a result, the AESO-THFA system in the presence of fillers showed the lowest values of E’ and Tg, revealing lower mechanical properties and the formation of very flexible polymers.

### 3.7. Tensile Properties

Finally, the tensile properties at room temperature of the photocured AESO-based specimens were investigated by tensile tests. Elastic modulus, tensile strength, and elongation at break values, in percentages, were determined from their stress–strain curves and are summarized in Table 4. The results of the tests indicate, as expected, that the tensile properties of the AESO-IBOMA and AESO-THFA samples at room temperature were totally different. The values of elastic modulus, tensile strength, and elongation at break of AESO-IBOMA were more than one order of magnitude higher than that of the AESO-THFA, and this was strictly dependent on the reactive diluent characteristics.

However, analyzing the values of the tensile properties of the AESO-based composites filled with the agro-wastes, it was possible to see that a significant increase in the Young’s modulus and tensile strength occurred for samples realized by the LCD 3D printer by adding the two agro-wastes fillers into the formulations prepared by mixing AESO with IBOMA as a reactive diluent. This could certainly have been due to the rigid iso-bornyl group which led the photo-crosslinked polymers to be stiffer and inflexible, also suggesting good adhesion among the printed layers [14]. In particular, the elastic modulus and the tensile strength values became double in the presence of 5 phr of GTF and WPL-CF, underlining the reinforcing effect induced by the biofiller powders within the polymeric resin. The filler particles could create a 3D network inside the matrix, enhancing the final mechanical response of the system [16]. The opposite trend occurred for the elongation at break that decreased, as is well known from the literature, from 12 wt.% to 5–6 wt.5% in the presence of biofillers [16].

A completely different situation can be seen for the AESO-THFA specimens. In this case, the AESO-THFA resins showed lower mechanical properties, giving rise to the formation of soft and flexible specimens. Furthermore, the addition of the biofillers did not affect the final mechanical properties in a positive way, which remained almost the same. This could most likely have been due to the presence of agglomerates of filler particles within the polymeric matrix, as already seen from SEM micrographs, leading to a decrease in the elasticity and tensile strength, increasing the polymer chain flexibility [14].

Considering the results obtained, it can be concluded that AESO-based polymers demonstrate tunable properties, varying from rigid to flexible, in the presence of different diluents (IBOMA and THFA) and biofillers (GTF and WPL-CF). The comparison of the tensile properties between the two series of specimens clearly evidenced that the AESO-IBOMA system gave rise to the best mechanical properties, suggesting that it was characterized by a very highly crosslinked structure and can be used with success for the realization of structural 3D-printed parts by LCD.

**Figure 11:**
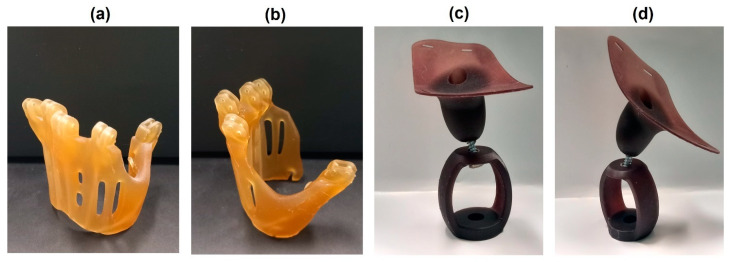
Three-dimensionally printed AESO-based components prepared with IBOMA for biomedical application, representing a human metacarpal hand prosthesis with GTF (**a**,**b**) and a pug dog prosthesis with WPL-CF (**c**,**d**).

Figure 11 reports two examples of components printed starting from the AESO-IBOMA system that can be used for biomedical applications.

The pictures in Figure 11a,b show two different views of a prosthesis for a human anatomy-based metacarpal hand designed to help people with missing fingers. The sample referred to the AESO-IBOMA+5GTF formulation. Figure 11c,d present a typical prosthesis for a pug dog that had completely lost a left front limb, realized by LCD with reference to the AESO-IBOMA+5WPL-CF formulation. The components show a very good level of definition and high dimensional stability.

## 4. Conclusions

The present work focused on the preparation of bio-based polymer composites by LCD as a 3D-printing process by means of the addition of two different agro-waste fillers, GTF and WPL-CF, obtained by corn and wine production wastes, within renewable soybean oil-based photocurable formulations, in the presence of two reactive diluents, IBOMA and THFA.

The comparison between the two kinds of resin systems, AESO-IBOMA and AESO-THFA, revealed significant differences. First, the zero-shear viscosity of the AESO-based photocurable formulations increased in the presence of agro-wastes for mixtures with IBOMA, reaching ideal values for printable resin in the vat polymerization 3D-printing process. Once the viscosity was evaluated and the optimal printing parameters were set, several 3D-printed components with different geometries and complexities were realized by LCD with and without fillers, such as gyroid cube, hexagon with intricate internal design, or hexagonal honeycomb-like cell structures made by the deposition of a high number of layers during the VP process. The composites prepared by adding the two different fillers at 5 phr within the polymeric matrix were fully characterized from the morphological, thermal, and mechanical points of view.

The results obtained revealed that agro-wastes’ addition into the AESO-based matrix induced a significant increase in the elastic modulus, tensile strength, and glass transition temperature values, especially for the mixtures containing IBOMA as the reactive diluent. This can be attributed to a stiffening effect induced to the presence of the filler particles which acted as reinforcing agents. The biofillers also gave rise to characteristic yellow and purple pigmentation in the specimens due to their dye action within the photocured resin. In contrast, the addition of the two fillers did not have an effect on the thermal stability of the composites, which showed almost the same thermal behavior. This finding allowed us to conclude that strong crosslinked structures can be obtained by LCD, giving rise to structural 3D-printed parts with very highly detailed architectures useful for applications in the biomedical field. A prosthesis for a human metacarpal hand, obtained by dispersing 5 phr of GTF within the AESO-IBOMA resin, and a pug dog prosthesis filled with 5 phr of WPL-CF powder were also successfully realized.

This study undoubtedly deserves further investigations to evaluate the biodegradability, cytotoxicity, and cell proliferation of the obtained photocured samples, as well as to optimize the 3D-printing process to develop even more complex 3D structures.

This research can significantly enhance the use of bio-based and renewable materials for the 3D printing of parts for biomedical applications using LCD as the vat polymerization technique. In addition, the use of biofillers can valorize agricultural wastes, reducing costs and promoting the development of a new generation of 3D-printed biodegradable composite materials with additional environmentally friendly impacts for the future.

## Figures and Tables

**Table 1:** Composition of photocurable AESO-based liquid formulations.

Sample Code	AESO (wt.%)	Diluent (wt.%)	Biofiller (phr)
AESO-IBOMA	60	40	0
AESO-IBOMA+5GTF	60	40	5
AESO-IBOMA+5WPL-CF	60	40	5
AESO-THFA	60	40	0
AESO-THFA+5GTF	60	40	5
AESO-THFA+5WPL-CF	60	40	5

**Table 2:** Printing parameters for AESO-based resins printed by LCD 3D printer.

Parameter	AESO-IBOMA	AESO-IBOMA +5GTF	AESO-IBOMA +5WPL-CF	AESO-THFA	AESO-THFA +5GTF	AESO-THFA +5WPL-CF
**Layer height** **(mm)**	0.1	0.1	0.1	0.1	0.1	0.1
**Exposure time** **(s)**	12	18	18	18	25	25
**Bottom exposure** **time (s)**	30	40	40	40	45	45
**Rest time before lift** **(s)**	0	3	3	0	5	5
**Rest time after lift** **(s)**	4	8	8	5	8	8
**Rest time after retract** **(s)**	3	6	6	3	6	6
**Bottom lift distance** **(mm)**	7	8	8	6	8	8
**Lifting distance** **(mm)**	6	8	8	6	8	8
**Bottom retract distance (mm)**	7	8	8	6	8	8
**Retract distance** **(mm)**	6	8	8	6	8	6
**Bottom lift speed** **(mm/min)**	60	50	50	90	70	70
**Lifting speed** **(mm/min)**	60	50	50	90	70	70
**Bottom retract speed (mm/min)**	150	130	130	170	150	150
**Retract speed** **(mm/min)**	150	130	130	170	150	150

**Table 3:** Gel %, thermal, and viscoelastic properties of AESO-based samples.

Sample	Gel (%)	T_onset_ (°C)	T_max deg_ (°C)	Ash (%)	E′@ 0 °C (MPa)	Tan Delta (°C)
AESO-IBOMA	99.5	297	320–380	1.5	1633	51
AESO-IBOMA + 5GTF	99.5	292	320–380	1.8	1855	80
AESO-IBOMA + 5WPL-CF	98.5	285	320–380	3.6	1846	76
AESO-THFA	99.9	304	393	1.0	1040	22
AESO-THFA + 5GTF	99.1	308	384	1.2	308	15
AESO-THFA + 5WPL-CF	99.6	296	378	2.7	309	12

**Table 4:** Tensile properties of AESO-based samples.

Sample	Young’s Modulus (MPa)	Tensile Strength (MPa)	Elongation at Break (%)
AESO-IBOMA	443 ± 25	16 ± 0.5	12 ± 3.0
AESO-IBOMA + 5GTF	987 ± 42	30 ± 0.1	6 ± 0.7
AESO-IBOMA + 5WPL-CF	933 ± 34	27 ± 1.6	5 ± 0.7
AESO-THFA	15 ± 1.0	3 ± 1.2	22 ± 9.0
AESO-THFA + 5GTF	15 ± 0.4	2 ± 0.3	13 ± 2.0
AESO-THFA + 5WPL-CF	15 ± 0.6	2 ± 0.3	15 ± 2.0

## Data Availability

Data are contained within the article.
